# Physical body experiences questionnaire simplified for active aging (PBE-QAG): Rasch validation

**DOI:** 10.1371/journal.pone.0280198

**Published:** 2023-02-10

**Authors:** Wei Deng, Sydney Carpentier, Ann Van de Winckel

**Affiliations:** 1 Division of Rehabilitation Science, Department of Rehabilitation Medicine, Medical School, University of Minnesota, Minneapolis, Minnesota, United States of America; 2 Division of Physical Therapy, Division of Rehabilitation Science, Department of Rehabilitation Medicine, Medical School, University of Minnesota, Minneapolis, Minnesota, United States of America; Mugla Sitki Kocman University: Mugla Sitki Kocman Universitesi, TURKEY

## Abstract

**Purpose:**

To validate the Physical Body Experiences Questionnaire simplified for Active aGing (PBE-QAG) with Rasch Measurement Theory. PBE-QAG measures body awareness during physical activity.

**Methods:**

Community-dwelling adults were recruited at the Minnesota State Fair, Highland Fest, and in the Brain Body Mind Lab (University of Minnesota). They completed demographic, clinical, and behavioral questionnaires and the PBE-QAG, which has 12 items, with scoring options ranging between 0 (totally true) and 4 (totally false). A lower total PBE-QAG score on reflects better body awareness. We validated the structural validity of PBE-QAG in community-dwelling older adults, and in community-dwelling adults (18–99 years of age). We also performed a pilot structural validity in community-dwelling adults with chronic stroke. We evaluated item and person fit, targeting, unidimensionality, person separation reliability, differential item functioning for demographic and clinical characteristics, principal component of residuals, and local item dependence.

**Results:**

We obtained unidimensionality and item fit after deleting and rescoring items in older adults (n = 133), adults (n = 530), and adults with chronic stroke (n = 36). In community-dwelling adults, 7 participants did not fit the model (1.13%). There was minimal floor (5.28%), no ceiling effect (0.00%), and no local item dependence or differential item functioning. The person mean location was -1.77±1.22 logits.

**Conclusions:**

PBE-QAG demonstrated good item and person fit, but the targeting is off. Therefore, the current version of PBE-QAG is not recommended for use in community-dwelling adults. We encourage further validation of PBE-QAG by adding more difficult items. We also recommend evaluating the PBE-QAG in a larger group of adults with stroke.

## Introduction

Physical activity as well as mind and body approaches help restore physical and mental health throughout the lifespan with at least two mechanisms: First, dysfunction in the autonomic nervous system is closely implicated at the onset and maintenance of disease states, and physical activity as well as mind and body approaches, help rebalance this dysfunction [1, 2]. Second, these practices are known to improve body awareness as well as the connection and awareness with and responsiveness to body sensations [3–7]. When adults are aware of their body in the present moment, they can also more easily notice physical or mental unease and thus take action to regain a healthier state [6].

This type of focus on the body is also called “internally oriented body awareness” (i.e., being more in tune with sensations and signals from the body as well as being more appreciative of the functionality of the body) in contrast to externally oriented body awareness (i.e., focusing on how the body appears to others) [8, 9]. One example of measuring internally oriented body awareness is through the physical body experience questionnaire (PBEQ) [10, 11]. The internally oriented body awareness leads to a better body appreciation and can be developed by performing regular physical activity [8–10]. In addition, yoga training, as well as regular physical training, have been associated with adults having a more positive relationship with their body [12, 13], and having a more conscious awareness of their physical skills and limitations [14]. The benefits of physical activity and mind and body approaches also extend to mental health consequences outside of disordered eating and body image disturbance and can lead to more positive health behaviors and increased self-esteem [10].

Originally, Menzel (2010) created an Athletic Body Experience Questionnaire, but then revised it to a PBEQ scale with 32 items and 7 scoring categories with an intent to use it in a broader population, not just for athletes [10, 11]. After testing this scale in a pilot study in 670 undergraduate female students, Menzel (2010) revised the PBEQ with fewer negatively worded items and shorter item descriptions. To avoid responder fatigue, the author reduced the scale to 18 items, which were related to Mind/Body Connection, Body Acceptance, Physical Competence, and Physical Limits. Menzel (2010) proposed that those 4 elements are key aspects of “embodiment” in physical activity, which is the unidimensional construct measured with PBEQ [11]. Embodiment is defined by Menzel (2010) as “*a connected*, *healthy*, *loving mind-body relationship*, *which encompasses the relationship between self and body*, *but also the experience of body function*, *bodily sensations*, *body awareness*, *and physical competence*” [11]. Both the exploratory and confirmatory factor analysis of this version of the scale suggested a single factor with a high internal consistency of Cronbach’s α = 0.94 [11]. The PBEQ correlated with other measures of embodiment: body awareness, body responsiveness, and body appreciation [11]. The PBEQ predicted various elements of embodiment, i.e., body responsiveness, body awareness, and positive body image, but also related to general mental well-being such as self-esteem [11]. Yet, while these results are promising, the scale was only tested in undergraduate female students. Also, the large number of scoring options (7) is usually problematic in terms of the ability to distinguish different levels of embodiment in participants [15].

Cossu et al. (2018) revised and simplified the PBEQ scale to identify positive internally oriented body awareness in relation to physical activity. This new Physical Body Experiences Questionnaire Simplified for Active Aging (PBE-QAG) is composed of 12 items [16]. Cossu et al. (2018) identified the same 4 key aspects of embodiment as previously presented by Menzel (2010) with internal consistency ranging from Cronbach’s α above 0.65 to 0.80, but given that his scale was a simplification of the original scale with rephrased statements, he defined the overall construct as “***body awareness related to physical activity***”. Cossu et al. (2018) further demonstrated that people who did more than 10 min of physical activity per week had a lower score on the PBE-QAG, reflecting a higher level of body awareness related to physical activity [16].

While those analyses give some indication of unidimensionality (confirmatory factor analyses), and internal reliability (Cronbach’s alpha), they are insufficient to encompass the psychometric rigor needed to promote a scale for use in the clinic or research. Using Rasch Measurement (RM) Theory, we transform an ordinal scale into an interval scale level of measurement, by accounting for both the participant’s level of ability on the investigated trait and the relative item difficulty [17, 18].

Therefore, as a next step in obtaining a more rigorous psychometric analysis of the PBE-QAG, the first aim of our study was to evaluate the structural validity and unidimensionality of PBE-QAG with RM Theory in community-dwelling older adults in the United States, given that the PBE-QAG was designed for this population. Next, given the importance of physical activity and mind and body practices for physical and mental well-being over the lifespan [19], our second aim was to evaluate the structural validity and unidimensionality of the PBE-QAG with RM Theory in community-dwelling adults, ages 18–99 years of age. Our third aim was to perform a preliminary RM analysis of the PBE-QAG in community-dwelling adults with chronic stroke, given that sensorimotor impairments often are accompanied by deficits in body awareness [20, 21].

## Materials and methods

### Participants

For this cross-sectional study, we recruited adults in the community through the University of Minnesota’s Driven to Discover research booth at the Minnesota State Fair and Highland Fest. Additionally, community-dwelling adults who previously had expressed interest in participating in research done at the Brain Body Mind Lab (University of Minnesota) were invited by email to participate in this study on evaluating body awareness and body image through questionnaires. The healthy adults at the State Fair and Highland Fest, as well as healthy adults who received an email, accessed the REDCap questionnaires through a secure REDCap web link. After reading and providing consent, they were quizzed on the comprehension of the content of the consent form through the University of California, San Diego Brief Assessment of Capacity to Consent (UBACC) [22]. They then completed the survey on a tablet (at the State Fair or Highland Fest) or on their computer. All data were automatically stored on the University of Minnesota’s Research Electronic Data Capture (REDCap). The REDCap uses a MySQL database via a secure web interface with data checks to ensure data quality during data entry. Access to REDCap study data is restricted to study team members by username and password. We did not collect any personal or identifiable information. We included participants between 18 and 99 years old. We excluded pregnant women and adults who did not speak English.

Additionally, adults with chronic stroke, who were involved in the development of a body awareness scale for adults with stroke, filled in the PBE-QAG as part of their assessment battery. They did not receive therapy but completed a one-time clinical assessment. They were recruited from the community via volunteer sampling using flyers and website announcements. Adults with stroke signed informed consent on paper and their assessments were collected on paper at their one-time assessment. The inclusion criteria were adults between 18 and 99 years of age, medically stable with an ischemic or hemorrhagic stroke and resulting left or right hemiplegia, longer than 6 months from stroke-onset, able to sign consent, able to hear, read and comprehend instructions given during the study, and be fluent in English.

The studies were approved by the University of Minnesota’s Institutional Review Board (IRB# STUDY00005849 and STUDY00000821) and were performed in accordance with the Declaration of Helsinki.

### Main outcome measures

The survey encompassed questions on demographical data, clinical information related to self-reported pain, self-reported mental health conditions, as well as behavioral questions on whether participants had past or current experience with practices of mindfulness, breathing exercises, or body awareness, such as yoga, Qigong, or Pilates. None of the participants were involved in a clinical trial or received any body awareness intervention as part of research. All reports were on practices that they would do on their own at home or in the community.

The PBE-QAG has 12 items with lower scores representing a higher level of body awareness related to physical activity. Participants responded to which extent those statements were true for them, by choosing a response between 1 (totally true) to 5 (totally false) with unlabeled interim integers. For the RM analysis, the scoring categories had to be renumbered from 1–5 to scoring categories 0–4.

### Sample size analysis

For RM analysis, sample sizes greater than 200 participants are recommended to accurately identify item fit [23]. Differential item functioning (DIF) can be investigated if the sample size exceeds 500 participants and the subgroups have a sample size of 200 [24].

### Statistical analysis

RM Theory is evaluating structural validity and unidimensionality. A scale is considered unidimensional when all items reflect the same underlying construct. RM Theory is based on a probabilistic model, which states that a person with a higher ability on a certain trait should have a higher probability of obtaining a better score (in the present context of PBE-QAG, a better score is a lower score). RM analysis transforms an ordinal scale into an interval scale with the ability of the persons and difficulty of the items represented on the same log-odds ratio (logits) units. We used the Partial Credit Model and analyzed the data with the Rasch Unidimensional Measurement Model (RUMM) 2030 software (RUMM Laboratory, Perth, WA, Australia).

To assess structural validity and unidimensionality, the RM analysis provides the following output: the presence of disordered thresholds, item and person fit, person separation reliability (PSR), mean error variance, targeting, the latter which includes floor- and ceiling effect, DIF, principal component analysis of residuals (PCAR), and local item dependence (LID). These terms are explained in more detail below.

*Disordered thresholds* of items indicate that the order of the response categories is not following the logically estimated order of the underlying construct [25, 26].

*Item- and person fit* statistics are reported as Residuals and Chi-square statistics, with Fit Residuals greater than +2.50 and less than -2.50 indicating item misfit, and item redundancy, respectively [17].

The *PSR* is a measure of precision that reflects how well persons can be differentiated into different levels of low and high ability of the trait to serve the clinical or research purpose [27]. To distinguish different ability levels among persons, this value should be at a minimum of 0.70 for group decisions; and over 0.90 to make decisions for individuals [17, 28, 29]. Another measurement of precision in the RM output is the mean error variance, reflecting the Root Mean Square standard Error computed over the persons or the items [30]. The Root Mean Square standard Error ranges from 0 to 1 with values closer to zero or lower than .30 indicating that the parameter estimate is more accurate [31].

Adequate *targeting* is obtained when the average person location is within a range of -0.50 and 0.50 logits of the average item location, which by default is set at 0 logits (and 1 logit standard deviation) [32]. An additional measure of targeting is floor-and ceiling effects. Floor- and ceiling effects become problematic when 15% or more of the sample obtains a minimum (floor) or maximum score (ceiling) on the assessment [33].

*DIF* evaluates whether the hierarchy of the items of the measurement is preserved across demographic or clinical factors [34]. A shift greater than 0.50 logits is considered evidence of DIF [35].

*PCAR* indicates the extent to which variance in the residuals is random. If so, then unidimensionality is implied because this variance is then explained by the underlying trait, and not by other underlying traits. PCAR’s output of the first contrast will reflect this with an eigenvalue of less than 2 and with the percent variance explained by this first contrast being less than 10% [36]. If this is not the case, then unidimensionality can be further tested by performing paired *t*-tests of 2 subtests of items that load respectively positively and negatively (correlations above and below 0.30) on the first contrast. Multidimensionality is noted when more than 5% of these *t*-tests are significant [17].

Finally, *LID* occurs when a pair of items shares a greater degree of content than with other items of the assessment. LID is reported for standardized residual item correlations that are at least 0.20 above the average residual item correlation [37]. For all analyses, where appropriate, Bonferroni corrected *p*-values are reported.

## Results

### Demographic and clinical data

We recruited in total 566 adults among which 36 were adults with chronic stroke resulting in left or right hemiplegia or hemiparesis of the upper limb. The 566 adults provided complete datasets. Table 1 displays the demographic and clinical data of each of those groups.

**Table 1:** Demographic and clinical characteristics of participants by group.

	Older adults without stroke (n = 133)	All adults without stroke (n = 530)	Adults with chronic stroke (n = 36)
**Age (years, mean±SD)**	70.35±4.82	50.09±16.82	58.00±12.93
**Gender**			
Female	77	338	11
Male	56	191	25
Other	0	1	0
**Level of education (years, mean±SD)**	5.98±1.81	5.85±1.84	5.56±1.66
**Ethnicity**			
Not Hispanic or Latino	132	522	36
Hispanic or Latino	1	8	0
**Racial background**			
American Indian or Alaska Native	0	2	0
Asian	3	30	2
Black/ African American	0	11	1
White	127	467	30
Multiracial	1	10	2
Other	2	10	1
**Pain**	34	108	18
**Mental health conditions**	37	204	2
**Current breathing exercise**	50	217	NA
**Current mindful relaxation exercise**	33	172	NA
**Current body awareness training**	44	170	NA

**Legend:** NA = not assessed

### Rasch Measurement Theory

The iteration analysis table (S1 Table) shows the step-by-step iteration of the RM analysis performed on the PBE-QAG for each group, i.e., community-dwelling older adults, community-dwelling adults, and adults with stroke. Per the iteration step, S1 Table displays the number of rating scales categories and number of items, person mean location in logits, mean error variance, floor, and ceiling effect, overall fit, item and person fit, number of items with disordered thresholds, PCAR, and PSR at every iteration step.

#### Community-dwelling older adults

**Fig 1:**
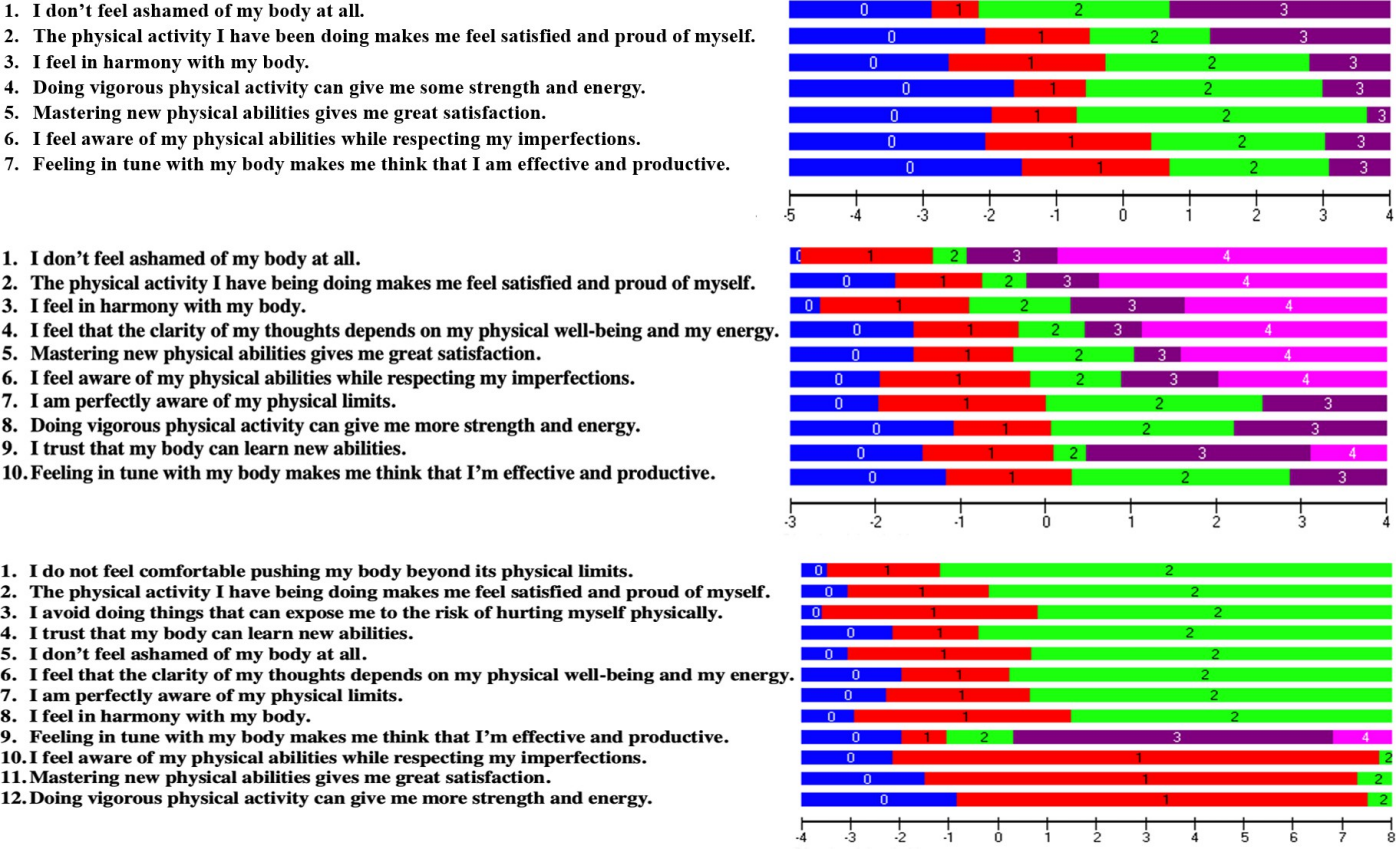
A. Item threshold map for community-dwelling older adults except stroke. B. Item threshold map for community-dwelling adults except stroke. C. Item threshold map for community-dwelling adults with chronic stroke.

The following iterations were performed: First, all items except item 5 “*I trust that my body can learn new abilities*” were rescored with scoring options [0 1 2 2 3] instead of [0 1 2 3 4]. Next, item 2 “*I avoid doing things that can expose me to the risk of hurting myself physically*” was rescored to [0 0 1 1 2]. Unfortunately, even after rescoring, those items still displayed item misfit (i.e., item 2, Fit Residual = 3.50, *p* = 0.001; item 11, Fit Residual = 3.96, *p<*0.0001. Item fit was obtained after deleting items 2, and item 11 *“I do not feel comfortable pushing my body beyond its physical limits*”. Item 3 “*I am perfectly aware of my physical limits*”; item 5 “*I trust that my body can learn new abilities*”; and item 12 “*I feel that the clarity of my thoughts depends on my physical well-being and my energy*” were subsequently removed to improve the targeting, as those items appeared to be too easy for this group. Table 2.1 in S2 Table shows the resulting item location in logits for each item after the above steps were completed in community-dwelling older adults without stroke. Table 2 shows the scoring categories of the revised PBE-QAG for community-dwelling older adults. The item threshold map (Fig 1A) displays the items hierarchy from the easiest item on the top to the hardest item on the bottom.

**Table 2:** Revised Rasch-based PBE-QAG for community-dwelling older adults.

Rasch-based PBE-QAG	Totally true				Totally false
I do not feel ashamed of my body at all.	0	1	2	2	3
The physical activity I have been doing makes me feel satisfied and proud of myself.	0	1	2	2	3
I feel in harmony with my body.	0	1	2	2	3
Doing vigorous physical activity can give me more strength and energy.	0	1	2	2	3
Mastering new physical abilities gives me great satisfaction.	0	1	2	2	3
I feel aware of my physical abilities while respecting my imperfections.	0	1	2	2	3
Feeling in tune with my body makes me think that I am effective and productive.	0	1	2	2	3

**Fig 2:**
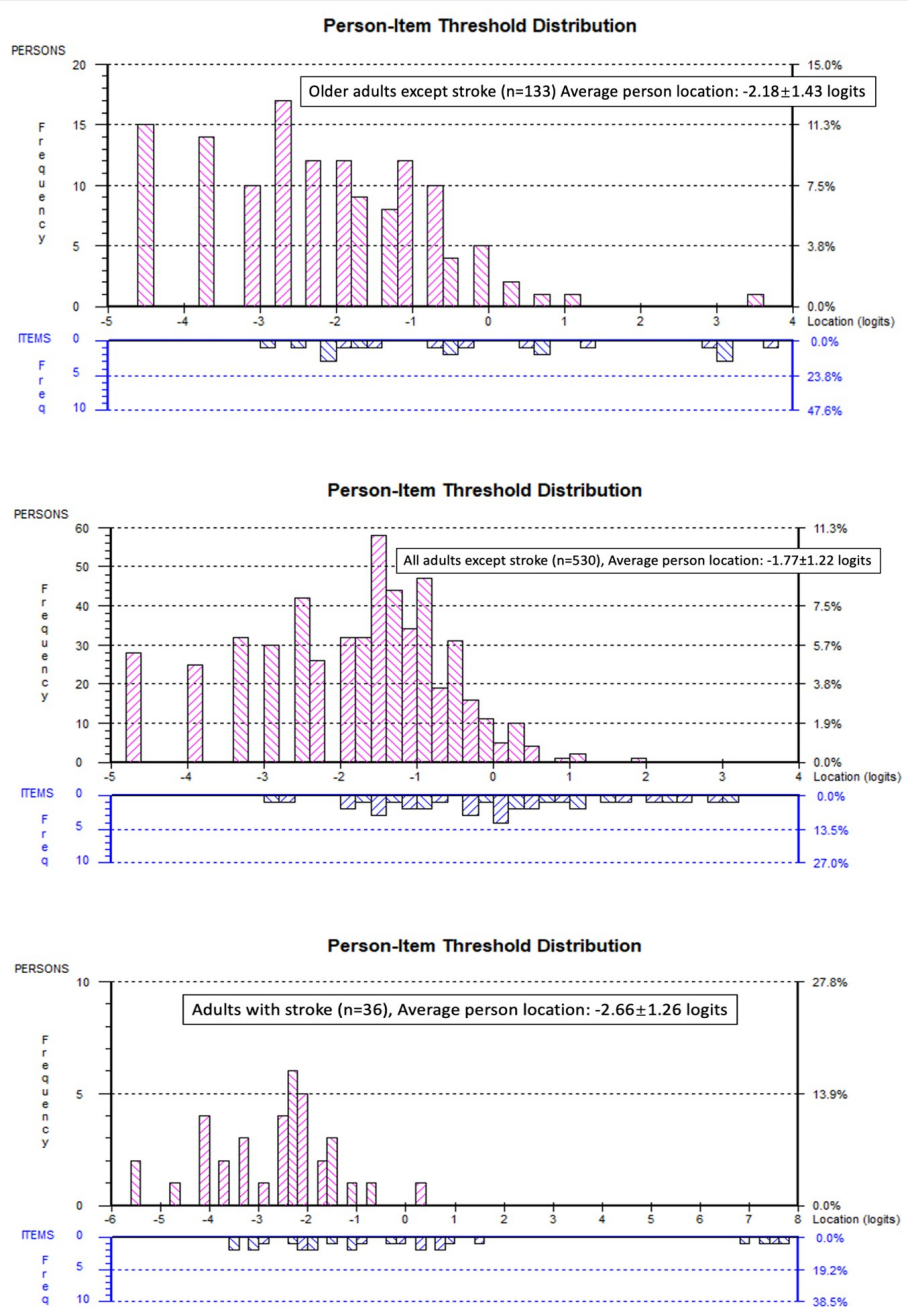
A. Person-Item threshold distribution for community-dwelling older adults except stroke. B. Person-Item threshold distribution for community-dwelling adults except stroke. C. Person-Item threshold distribution for community-dwelling adults with chronic stroke.

The person fit was excellent with only 1 person out of 133 (0.75%) displaying misfit. PSR was 0.72 and thus the scale would have acceptable reliability to make group decisions. The mean error variance was 0.56 logits. However, even though floor (11.28%) was not problematic and there was no ceiling effect (0.00%), the targeting for this older adult group was off: the person mean location was -2.18±1.43 logits (Fig 2A), meaning that the items were too easy for this group and thus we do not recommend using this scale for older adults in its present form.

The item threshold map shows the item hierarchy in terms of item difficulty, with the easiest item on top (“*I do not feel ashamed of my body at all”*) and the hardest item on the bottom (“*Feeling in tune with my body makes me think that I am effective and productive”*). The location of the thresholds between the scoring categories is represented on the logit scale at the bottom (the horizontal black line). This horizontal black line (interval logit scale) also represents the level of body awareness related to physical activity that participants have, with a lower score reflecting a higher body awareness level.

The pink histograms represent the frequency of participants at their body awareness ability level from a high level of body awareness (lowest logit value on the left side of the scale) to a low level of body awareness (highest logit value on the right side of the scale). The frequency of item thresholds is represented at the bottom of the figure (diagonal blue stripes histogram) on the same logit scale, organized from the lowest scoring threshold categories on the left to the highest scoring threshold categories of items on the right. A lower score reflects a higher body awareness level.

Given that DIF is calculated for variables that have subgroups of n = 200 or greater [38, 39], we did not perform DIF for this subgroup of older adults. Even though the PCAR eigenvalue was 1.63 with 23.34% variance explained by this first contrast, the paired *t*-tests revealed that only 3.01% of persons had significantly different person locations among the two subsets of items, thereby supporting the unidimensionality of this scale. No consequential LID was found.

#### Community-dwelling adults

We collected data from 530 community-dwelling adults (excluding adults with chronic stroke).

First, five items were rescored to scoring options [0 1 2 2 3] instead of [0 1 2 3 4] because of reversed thresholds. These five items were: item 2; item 3; item 5; item 7 “*Doing vigorous physical activity can give me more strength and energy*”; and item 11.

Next, item 2 (Fit Residual = 6.21; *p*<0.0001), and item 11 (Fit Residual = 6.90; *p*<0.0001) were deleted because of misfit. After deleting items 11 and 2, item 1 “*I do not feel ashamed of my body at all*” displayed misfit (Fit Residual = 3.78, *p* = 0.00012), but removing item 1 worsened the targeting (person mean location: -1.95±1.28 logits), as well as the PSR value (down to 0.75), and thus we decided to keep this item in the scale. Table 4 shows the scoring categories of the Rasch-based PBE-QAG for community-dwelling adults, taking into account the revisions proposed by the RM analysis. The item threshold map (Fig 1B) displays the items hierarchy from the easiest item on the top to the hardest item on the bottom. Table 2.2 in S2 Table shows the resulting item location in logits for each item after the above steps were completed in community-dwelling adults without stroke. Table 3 shows the scoring categories of the revised PBE-QAG for community-dwelling adults.

**Table 3:** Revised Rasch-based PBE-QAG for all community-dwelling adults (without adults with stroke).

Rasch-based PBE-QAG	Totally true				Totally false
I do not feel ashamed of my body at all.	0	1	2	3	4
The physical activity I have been doing makes me feel satisfied and proud of myself.	0	1	2	3	4
I feel in harmony with my body.	0	1	2	3	4
I feel that the clarity of my thoughts depends on my physical well-being and my energy.	0	1	2	3	4
Mastering new physical abilities gives me great satisfaction.	0	1	2	3	4
I feel aware of my physical abilities while respecting my imperfections.	0	1	2	3	4
I am perfectly aware of my physical limits.	0	1	2	2	3
Doing vigorous physical activity can give me more strength and energy.	0	1	2	2	3
I trust that my body can learn new abilities.	0	1	2	3	4
Feeling in tune with my body makes me think that I am effective and productive.	0	1	2	2	3

**Table 4:** Revised Rasch-based PBE-QAG for adults with chronic stroke.

Rasch-based PBE-QAG	Totally true				Totally false
1. I do not feel comfortable pushing my body beyond its physical limits.	0	1	1	1	2
2. The physical activity I have been doing makes me feel satisfied and proud of myself.	0	1	1	1	2
3. I avoid doing things that can expose me to the risk of hurting myself physically.	0	1	1	1	2
4. I trust that my body can learn new abilities.	0	1	1	1	2
5. I do not feel ashamed of my body at all.	0	1	1	1	2
6. I feel that the clarity of my thoughts depends on my physical well-being and my energy.	0	1	1	1	2
7. I am perfectly aware of my physical limits.	0	1	1	1	2
8. I feel in harmony with my body.	0	1	1	1	2
9. Feeling in tune with my body makes me think that I am effective and productive.	0	1	2	3	4
10. I feel aware of my physical abilities while respecting my imperfections.	0	1	1	1	2
11. Mastering new physical abilities gives me great satisfaction.	0	1	1	1	2
12. Doing vigorous physical activity can give me more strength and energy.	0	1	1	1	2

The person fit was excellent with only 7 persons out of 530 (18.06%) displaying misfit. PSR was 0.78 and thus the scale would have acceptable reliability to make group decisions. The mean error variance was 0.32 logits. Twenty-eight adults scored a minimum value (floor effect, 5.28%), and no one scored maximum (ceiling effect, 0.00%), but we also encountered poor targeting in the larger adult group with the person mean location being -1.77±1.22 logits (Fig 2B), meaning that the items are too easy for this community-dwelling adults and, thus, we recommend improving the targeting of this scale prior to use of this scale in the community.

The item threshold map shows the item hierarchy in terms of item difficulty, with the easiest item on top and the hardest item on the bottom. The location of the thresholds between the scoring categories is represented on the logit scale at the bottom (the horizontal black line). This horizontal black line (interval logit scale) also represents the level of body awareness related to physical activity that participants have, with a higher score reflecting a lower body awareness level.

The pink histograms represent the frequency of participants at their body awareness ability level from a high level of body awareness (lowest logit value on the left side of the scale) to a low level of body awareness (highest logit value on the right side of the scale). The frequency of item thresholds is represented at the bottom of the figure (diagonal blue stripes histogram) on the same logit scale, organized from the lowest scoring threshold categories on the left to the highest scoring threshold categories of items on the right. A lower score reflects a higher body awareness level.

There was no DIF for sex, persons with or without mental health conditions, or whether participants performed breathing or body awareness training. This means that the scale structure is preserved across these subgroups. The PCAR eigenvalue was 1.75 with a percent variance of 19.38%, but only 4.72% of persons had significantly different person locations among the two subsets of items, supporting the unidimensionality of this scale. No LID was found.

#### Adults with chronic stroke

Finally, our pilot RM analysis in 36 adults with chronic stroke revealed all items except 1 item “*Feeling in tune with my body makes me think that I am effective and productive*” had to be rescored to [0 1 1 1 2] to address the reversed thresholds. The item threshold map (Fig 1C) displays the items hierarchy from the easiest item on the top to the hardest item on the bottom. The resulting 12 items scale with rescored item categories revealed no item or person misfit, a PSR of 0.70, and a person mean location of -2.66±1.26 logits (Fig 2C), demonstrating that also for this group, the items were too easy, although a larger sample would be needed to confirm these findings. Table 4 shows the scoring categories of the Rasch-based PBE-QAG for community-dwelling adults, taking into account the revisions proposed by the RM analysis. Table 2.3 in S2 Table shows the resulting item location in logits for each item after rescoring the items.

We tested out whether deleting 4 items would improve the targeting, as they seemed too easy for this group: i.e., original item 6 “*Feeling in tune with my body makes me think that I’m effective and productive*”, item 7, item 8 “*I feel aware of my physical abilities while respecting my imperfections*”, and item 10 “*Mastering new physical abilities gives me great satisfaction*”, the targeting was improved (person mean location = -1.32±1.14, but it also reduced the PSR to an unacceptable level (PSR = 0.50) so we decided to leave those items in.

The item threshold map shows the item hierarchy in terms of item difficulty, with the easiest item on top and the hardest item on the bottom. The location of the thresholds between the scoring categories is represented on the logit scale at the bottom (the horizontal black line). This horizontal black line (interval logit scale) also represents the level of body awareness related to physical activity that participants have, with a higher score reflecting a lower body awareness level.

The pink histograms represent the frequency of participants at their body awareness ability level from a high level of body awareness (lowest logit value on the left side of the scale) to a low level of body awareness (highest logit value on the right side of the scale). The frequency of item thresholds is represented at the bottom of the figure (diagonal blue stripes histogram) on the same logit scale, organized from the lowest scoring threshold categories on the left to the highest scoring threshold categories of items on the right. A lower score reflects a higher body awareness level.

Given that this is a pilot RM analysis in a limited number of adults with stroke (n = 36), we did not perform DIF. Even though the PCAR eigenvalue was 2.02 with a percent variance explained by this first contrast of 16.82%, the paired *t*-tests revealed that that 5.56% of persons had significantly different person locations among the two subsets of items, which is above the 5% limit to support the unidimensionality of this scale, but given the small sample size, these results need to be validated in a larger sample of adults with chronic stroke. No consequential LID was found.

## Discussion

The present study demonstrates a Rasch-based validation of the PBE-QAG scale, which measures body awareness during physical activity. The first aim of our study was to evaluate the structural validity and unidimensionality of the PBE-QAG with RM Theory in community-dwelling older adults. Next, we evaluated the structural validity and unidimensionality of the PBE-QAG with RM Theory in community-dwelling adults (18–99 years old). Finally, we performed a preliminary RM analysis of the PBE-QAG in community-dwelling adults with chronic stroke.

The analyses in the community-dwelling older adults and the larger group of adults revealed that rescoring and deleting items were required. One of the reasons rescoring may have been needed could be because the intermediate scoring options were not labeled [40, 41]. Numeric labels that only contain end-form verbal explanations tend to have lower reliability because the unlabeled integers implicate uncertainty, and participants have to rely on their own labeling system to decide which scoring category to select [40]. Our study seems to confirm this assertion given that the middle scores needed to be merged.

In both analyses, items 2 and 11 needed to be deleted. These items belonged to the subgroup of “physical limitations”. This subgroup of statements about physical limitations did not obtain good internal consistency in earlier studies that reported on factor analysis [11, 16]. Even though deleting items 3, 5, and 12 improved targeting in the older adult group, the remaining 7 items still demonstrated poor targeting, despite that the PBE-QAG was originally designed for older adults [16]. Cossu et al. (2018) designed the scale such that a lower score represents a better ability [16]. In the group of older adults in our study, the person mean location was -2.18±1.43, which demonstrates that the item difficulty of this scale is too easy for this group. Additionally, this results in poor measurement precision for the persons with the lowest body awareness levels related to physical activity (at the right-hand side of the logit ruler). We encountered a similar poor targeting in the larger group of community-dwelling adults with a person mean location of -1.77±1.22. A solution to improve targeting and precision and the right end of the scale could be to add more difficult items and re-evaluate the structural validity, unidimensionality, and especially the targeting in a new RM analysis in community-dwelling adults.

Cossu et al. (2018) noticed that older adults who were doing regular physical activity had a higher body awareness related to physical activity [16]. Similar observations have been made with regard to mind and body approaches [6]. The latter seems to be the case in our groups as well. Our descriptive analysis showed that persons who are performing body awareness training in daily life appear to obtain a lower score (and thus better body awareness) compared to those who do not perform such training. The person mean locations were -3.18±1.44 vs -2.58±1.36 for the body awareness group vs no body awareness respectively, in the older adults; and -2.12±1.26 vs -1.60±1.17 in all adults.

After rescoring and deleting items, the PBE-QAG showed adequate fit statistics and evidence of unidimensionality for older adults (PBE-QAG with 7 items) as well as for the larger group of community-dwelling adults. (PBE-QAG with 10 items). Unidimensionality was supported by the PCAR’s paired *t*-tests, confirming that the remaining 7 items and 10 items, respectively, were measuring the same construct of “body awareness related to physical activity”.

Both analyses demonstrated adequate reliability, minimal floor and no ceiling effect, and no local dependency. Additionally, the larger adult group also demonstrated that there was no DIF for sex, persons with or without mental health conditions, or whether participants performed breathing or body awareness training, meaning that the hierarchy of the measurement was preserved across these demographic and behavioral subgroups.

Our preliminary analysis in adults with stroke showed that we could achieve item and person fit by reducing the scoring categories to 3 options in all but 1 item (item “*Feeling in tune with my body makes me think that I am effective and productive*"), which had ordered thresholds while keeping the original scoring categories. However, a larger sample is needed to identify targeting and unidimensionality.

### Study limitations

Although we obtained a large sample size overall of community-dwelling adults, our sample size of adults with chronic stroke was smaller. Therefore, a RM validation in a larger sample of adults with chronic stroke would be needed to ascertain the targeting and structural validity of the PBE-QAG in adults with chronic stroke. Second, we recruited most of the participants at the State Fair through convenience sampling, which might have induced some selection bias. Third, given that the State of Minnesota has a 78.1% White non-Hispanic population [42] our samples lacked diversity. Future research should be more inclusive and include a larger sample of adults from diverse backgrounds. One solution could be to recruit nationally, given that the measurement was provided online through a REDCap link.

## Conclusions

The Rasch-based PBE-QAG demonstrates good item and person fit as well as unidimensionality, but the targeting leaves room for improvement. We recommend adding items that target more complex aspects of body awareness related to physical activity might be needed as well as labeling the intermediate integers, followed by a new RM validation to test targeting, structural validity, and unidimensionality in community-dwelling adults. Additionally, other psychometric analyses need to be performed such as test-retest reliability, sensitivity to change, etc. before this scale can be used for assessments in intervention studies or in the clinic.

## Supporting information

S1 Table Iterations analysis table of the PBE-QAG. DF = Degrees of Freedom; PCAR = Principal Component Analysis of Residuals; PSR = Person Separation Reliability; RMSE = Root Mean Square Error (reflected as Mean Error Variance in RUMM2030).
(DOCX)

S2 Table Table 2.1. Item fit statistics of the Revised Physical Body Experiences Questionnaire Simplified for Active Aging (PBE-QAG) for older adults without stroke. Table 2.2. Item fit statistics of the Revised PBE-QAG for all adults without stroke. Table 2.3. Item fit statistics of the Revised PBE-QAG for adults with chronic stroke. Legend: SE = Standard Error.
(DOCX)

S1 Text (TXT)

S1 Data (CSV)

## Abbreviations

DIF: Differential Item Functioning
LID: Local Item Dependence
PBE-QAG: Physical Body Experiences Questionnaire Simplified for Active Aging
PCAR: Principal Component Analysis of Residuals
PSR: Person Separation Reliability
RM: Rasch Measurement
RUMM2030: Rasch Unidimensional Measurement Model 2030 software
