# Supplementary material for: Physical body experiences questionnaire simplified for active aging (PBE-QAG): Rasch validation
Source: PLoS One. 2023 Feb 10;18(2):e0280198. doi: 10.1371/journal.pone.0280198 (PMC9916545; doi:10.1371/journal.pone.0280198)
Supplement: S1 Table — DF = Degrees of Freedom; PCAR = Principal Component Analysis of Residuals; PSR = Person Separation Reliability; RMSE = Root Mean Square Error (reflected as Mean Error Variance in RUMM2030). (DOCX) [file pone.0280198.s001.docx]

**S1. Iteration analysis table**

| **Analysis** | **Items** | **Rating scale categories** | **Person mean (SD) logits** | **Mean error variance** | **Floor effect**  **n (%)** | **Ceiling effect**  **n (%)** | **Overall Chi-square (DF)**  **p-value** | **PSR** | **Items with disordered thresholds**  **(n)** | **Misfitting items**  **(n)** | **PCAR**  **Eigenvalue**  **1^st^ contrast**  **(%)** | **Misfitting persons**  **n (%)** |
| --- | --- | --- | --- | --- | --- | --- | --- | --- | --- | --- | --- | --- |
| **All items (n=133) older adults except stroke** | 12 | 60 | -1.80 (0.96) | 0.22 | 5 (3.76%) | 0  (0.00%) | 181.35 (98)  *p*<0.0001 | 0.73 | 11 | 1  (item 11) | 2.70 (22.43%) | 2 (1.50%) |
| All items (n=133)  Rescore 11 items [0-1-2-2-3] | 12 | 49 | -2.12  (1.08) | 0.26 | 5 (3.76%) | 0  (0.00%) | 161.40  (96)  *p*=0.00004 | 0.76 | 1  (item 2) | 1  (item 11) | 2.44  (20.29%) | 2 (1.50%) |
| All items (n=133)  Rescore item 2  [0-0-1-1-2] | 12 | 48 | -2.19  (1.10) | 0.28 | 5 (3.76%) | 0  (0.00%) | 162.45  (96)  *p*=0.00003 | 0.76 | 0 | 1  (item 11) | 2.27  (18.89%) | 2 (1.50%) |
| Item 11 deleted (n=133) | 11 | 44 | -2.48  (1.25) | 0.36 | 7  (5.26%) | 0  (0.00%) | 140.12  (88)  *p*=0.0004 | 0.77 | 0 | 1  (item 2) | 1.74  (15.81%) | 2 (1.50%) |
| Item 2 deleted (n=133) | 10 | 41 | -2.78  (1.41) | 0.43 | 10  (7.52%) | 0  (0.00%) | 115.78  (64)  *p*=0.00008 | 0.78 | 0 | 0 | 1.75  (17.48%) | 2 (1.50%) |
| Items 3,5,12  deleted  (n=133) | 7 | 21 | -2.18  (1.43) | 0.56 | 15  (11.28%) | 0  (0.00%) | 47.19  (56)  *p*=0.79 | 0.72 | 0 | 0 | 1.63  (23.34%) | 1  (0.75%) |
| **All items**  **(n=530)**  **All adults except stroke** | 12 | 60 | -1.12  (0.87) | 0.17 | 11  (2.08%) | 0  (0.00%) | 551.41  (96)  *p*<0.0001 | 0.75 | 5 | 2  (items 2, 11) | 2.50 (20.84%) | 10  (1.89%) |
| All items  (n=530)  5 items rescored to 0 1 2 2 3 | 12 | 55 | -1.34 (0.94) | 0.19 | 11  (2.08%) | 0  (0.00%) | 400.59  (96)  *p*<0.0001 | 0.76 | 0 | 2  (items 2, 11) | 2.20  (18.30%) | 8  (1.51%) |
| Item 11 deleted (n=530) | 11 | 51 | -1.53  (1.05) | 0.24 | 15  (2.83%) | 0  (0.00%) | 360.55 (88)  *p*<0.0001 | 0.77 | 0 | 1  (item 2) | 1.81  (16.45%) | 7  (1.32%) |
| Item 2 deleted (n=530) | 10 | 47 | -1.77 (1.22) | 0.32 | 28 (5.28%) | 0  (0.00%) | 167.69  (80)  *p*<0.0001 | 0.78 | 0 | 1  (item 1) | 1.81 (18.06%) | 7  (1.32%) |
| **All items**  **(n=36)**  **Adults with stroke** | 12 | 60 | -1.54  (0.85) | 0.20 | 2 (5.56%) | 0  (0.00%) | 89.87  (96)  *p=*0.66 | 0.67 | 11 | 0 | 2.10  (17.51%) | 0  (0.00%) |
| All items  (n=36)  Rescore 11 items  [0-1-1-1-2] | 12 | 38 | -2.66  (1.26) | 0.42 | 2 (5.56%) | 0  (0.00%) | 64.83  (72)  *p=*0.71 | 0.70 | 0 | 0 | 2.02  (16.82%) | 0  (0.00%) |

Legend: DF=Degrees of Freedom; PCAR=Principal Component Analysis of Residuals; PSR=Person Separation Reliability; RMSE=Root Mean Square Error (reflected as Mean Error Variance in RUMM2030)
