# Supplementary material for: Physical body experiences questionnaire simplified for active aging (PBE-QAG): Rasch validation
Source: PLoS One. 2023 Feb 10;18(2):e0280198. doi: 10.1371/journal.pone.0280198 (PMC9916545; doi:10.1371/journal.pone.0280198)
Supplement: S2 Table — Item fit statistics of the Revised Physical Body Experiences Questionnaire Simplified for Active Aging (PBE-QAG) for older adults without stroke. Table 2.2. Item fit statistics of the Revised PBE-QAG for all adults without stroke. Table 2.3. Item fit statistics of the Revised PBE-QAG for adults with chronic stroke. Legend: SE = Standard Error. (DOCX) [file pone.0280198.s002.docx]

**Table 2.1 Item fit statistics of the Revised Physical Body Experiences Questionnaire Simplified for Active Aging (PBE-QAG) for older adults without stroke**

| **Revised item number of PBE-QAG** | **Item location  (logits)** | **SE** | **Fit Residuals** | ***p*-value** |
| --- | --- | --- | --- | --- |
| 1. I do not feel ashamed of my body at all (e.g., I often wear clothes that enhance me, or I have no problem showing myself in a swimsuit at the sea or pool). | -1.43 | 0.13 | 1.71 | 0.14 |
| 2. The physical activity I have been doing makes me feel satisfied and proud of myself. | -0.41 | 0.14 | -0.06 | 0.68 |
| 3. I feel in harmony with my body (e.g., I like taking care of my body and knowing what kind of things can be good for me or not). | -0.02 | 0.16 | 0.03 | 0.89 |
| 4. Doing vigorous physical activity can give me some strength and energy. | 0.28 | 0.15 | 0.32 | 0.54 |
| 5. Mastering new physical abilities gives me great satisfaction (e.g., doing activities I have never done before). | 0.34 | 0.15 | -0.57 | 0.74 |
| 6. I feel aware of my physical abilities while respecting my imperfections. | 0.47 | 0.17 | -0.47 | 0.77 |
| 7. Feeling in tune with my body makes me think that I am effective and productive (e.g., finishing activities I suppose to be possible for me makes me feel good). | 0.77 | 0.17 | -0.89 | 0.38 |

**Legend:** SE = Standard Error

**Table 2.2 Item fit statistics of the Revised PBE-QAG for all adults without stroke**

| **Revised item number of PBE-QAG** | **Item location  (logits)** | **SE** | **Fit Residuals** | ***p*-value** |
| --- | --- | --- | --- | --- |
| 1. I do not feel ashamed of my body at all (e.g., I often wear clothes that enhance me, or I have no problem showing myself in a swimsuit at the sea or pool. | -1.24 | 0.05 | 3.78 | 0.0001 |
| 2. The physical activity I have being doing makes me feel satisfied and proud of myself. | -0.52 | 0.05 | -0.23 | 0.62 |
| 3. I feel in harmony with my body (e.g., I like taking care of my body and knowing what kind of things can be good for me or not). | -0.40 | 0.06 | -2.38 | 0.003 |
| 4. I feel that the clarity of my thoughts depends on my physical well-being and my energy (e.g., I find that by doing regular physical activity I am mentally more active). | -0.06 | 0.06 | 2.38 | 0.15 |
| 5. Mastering new physical abilities gives me great satisfaction (e.g., doing activities I‘ve never done before) | 0.18 | 0.06 | -0.22 | 0.47 |
| 6. I feel aware of my physical abilities while respecting my imperfections. | 0.20 | 0.07 | -2.58 | 0.003 |
| 7. I am perfectly aware of my physical limits (e.g., I know what kind of activities can be dangerous for my body). | 0.21 | 0.07 | 2.50 | 0.02 |
| 8. Doing vigorous physical activity can give me more strength and energy. | 0.41 | 0.07 | 0.11 | 0.90 |
| 9. I trust that my body can learn new abilities (activities that I have never done before). | 0.56 | 0.07 | -0.17 | 0.17 |
| 10. Feeling in tune with my body makes me think that I am effective and productive (e.g., finishing activities I suppose to be possible for me makes me feel good). | 0.67 | 0.08 | -3.18 | 0.0002 |

**Legend:** SE = Standard Error

**Table 2.3 Item fit statistics of the Revised PBE-QAG for adults with chronic stroke**

| **Revised item number of PBE-QAG** | **Item location  (logits)** | **SE** | **Fit Residuals** | ***p*-value** |
| --- | --- | --- | --- | --- |
| 1. I do not feel comfortable pushing my body beyond its physical limits (e.g., doing activities I suppose can be beyond my reach). | -2.32 | 0.29 | 1.05 | 0.54 |
| 2. The physical activity I have being doing makes me feel satisfied and proud of myself. | -1.61 | 0.32 | 0.94 | 0.57 |
| 3. I avoid doing things that can expose me to the risk of hurting myself physically (e.g., making excessive efforts, lifting weights too heavy for me). | -1.38 | 0.37 | -0.11 | 0.74 |
| 4. I trust that my body can learn new abilities (activities that I have never done before). | -1.26 | 0.30 | -0.22 | 0.29 |
| 5. I do not feel ashamed of my body at all (e.g., I often wear clothes that enhance me, or I have no problem showing myself in a swimsuit at the sea or pool. | -1.18 | 0.34 | 0.87 | 0.36 |
| 6. I feel that the clarity of my thoughts depends on my physical well-being and my energy (e.g., I find that by doing regular physical activity I am mentally more active). | -0.85 | 0.33 | 0.69 | 0.40 |
| 7. I am perfectly aware of my physical limits (e.g., I know what kind of activities can be dangerous for my body). | -0.81 | 0.34 | -0.17 | 0.90 |
| 8. I feel in harmony with my body (e.g., like taking care of my body and knowing what kind of things can be good for me or not). | -0.72 | 0.36 | -0.68 | 0.25 |
| 9. Feeling in tune with my body makes me think that I am effective and productive (e.g., finishing activities I suppose to be possible for me makes me feel good). | 1.03 | 0.26 | -1.03 | 0.71 |
| 10. I feel aware of my physical abilities while respecting my imperfections. | 2.81 | 0.38 | -1.29 | 0.15 |
| 11. Mastering new physical abilities gives me great satisfaction (e.g., doing activities I‘ve never done before). | 2.92 | 0.41 | 0.78 | 0.66 |
| 12. Doing vigorous physical activity can give me more strength and energy. | 3.36 | 0.47 | -0.70 | 0.68 |

**Legend:** SE = Standard Error
